# Enhancing Phycoerythrin Production of Marine Red Microalga *Porphyridium purpureum* with Low Salinity and Semi-Continuous Culture Strategy

**DOI:** 10.3390/md23090361

**Published:** 2025-09-19

**Authors:** Chulin Li, Houbo Wu, Hualian Wu, Wenzhou Xiang, Jin Xu, Tao Li

**Affiliations:** 1 State Key Laboratory of Tropical Oceanography, Guangdong Key Laboratory of Marine Materia Medica, South China Sea Institute of Oceanology, Chinese Academy of Sciences, Guangzhou 510301, China; lchlxpy@126.com (C.L.); wuhoubo@scsio.ac.cn (H.W.); hlwu@scsio.ac.cn (H.W.); xwz@scsio.ac.cn (W.X.); 2 Guangzhou Institute of Energy Conversion, Chinese Academy of Sciences, CAS Key Laboratory of Renewable Energy, Guangdong Provincial Key Laboratory of New and Renewable Energy Research and Development, Guangzhou 510640, China

**Keywords:** *Porphyridium*, phycoerythrin, exopolysaccharides, salinity, semi-continuous culture

## Abstract

*Porphyridium purpureum* can synthesize a high phycoerythrin content, which has strong potential application in nutrition, pharmaceuticals, and cosmetics. An effective culture strategy is the key to producing biomass of *P. purpureum* rich in phycoerythrin. However, there are still bottlenecks in the large-scale production of *Porphyridium*, such as nutrient supplementation and cultivation mode. In this study, *P. purpureum* SCS-02, isolated from the South China Sea, was used as experimental microalga strain. The effects of different salinity (10, 20 and 34 ppt) and semi-continuous culture on accumulation of biomass and phycoerythrin were investigated. The semi-continuous culture modes include recycled culture medium mode (RM) and fresh culture medium mode (FM). The results showed that low salinity (10 ppt) could enhance the accumulation of phycoerythrin, the content and yield of which were 8.39% DW and 160 mg L^−1^, respectively. The yield of phycoerythrin of *P. purpureum* in semi-continuous culture with a 30% renewal rate of fresh culture medium was 253% higher than the batch culture. In summary, the semi-continuous culture strategy with fresh medium renewal under low salinity conditions increased the phycoerythrin yield to 641.47 mg after 30 days of cultivation, while exopolysaccharide accumulation was significantly reduced compared with batch culture. These results provide useful reference for optimizing culture strategies of *P. purpureum*, and may serve as a basis for future attempts to scale phycoerythrin production under industrially relevant conditions.

## 1. Introduction

Phycoerythrin is one of the light-harvesting phycobiliproteins and can be mainly classified into three main types: R-phycoerythrin (R-PE), C-phycoerythrin (C-PE), and B-phycoerythrin (B-PE) [1]. R-PE is primarily derived from red macroalgae such as *Porphyra* sp. and *Gracilaria* sp., while C-PE is mainly found in cyanobacteria such as *Halomicronema* sp. [2]. Meanwhile, B-PE is predominantly produced by unicellular red microalgae *Porphyridium* sp., which is particularly notable for its high accumulation capacity [3]. *Porphyridium* can accumulate high levels of B-phycoerythrin, reaching up to 8.18% of dry weight (DW) in ASW medium [4] and 4.16% DW in F/2 medium [5]. B-phycoerythrin has attracted considerable interest due to its potential applications as a natural pigment [6] and in the pharmaceutical [7] and cosmetic industries [8]. Its synthesis is known to be significantly influenced by environmental and nutritional factors, including light [9], carbon [10], and nitrogen sources [11]. Nevertheless, large-scale microalgal cultivation still faces two major sustainability challenges: water footprint and nutrient utilization [12]. Addressing these issues requires strategies that reduce freshwater demand and improve nutrient efficiency. In this study, we focus on the influence of salinity and cultivation mode on phycoerythrin production in *Porphyridium*. Approaches such as semi-continuous cultivation and the use of low-salinity media may provide feasible solutions, thereby enhancing the potential of *Porphyridium* for sustainable large-scale applications.

Salinity is a crucial culture condition influencing the growth and pigment synthesis of microalgae [13]. Changes in the salinity of the culture medium can trigger a series of metabolic adjustments in microalgae. For example, microalgae can rapidly accumulate compatible solutes such as glycerol, floridoside, and glucosylglycerol [14,15] or adjust the photosynthetic apparatus to adapt to the osmotic pressure changes. It has been reported in *P. purpureum* that increasing salinity decreases the proportion of xylose and galactose while increasing the proportion of glucose [16]. Cryptophytes such as *Rhodomonas salina* have high phycoerythrin content in the salinity range from 30−40 ppt [17,18]. In *P. purpureum*, the effects of a wide salinity range (0, 17, 34, and 68 ppt) have been reported [19]. However, the impact of narrower salinity differences within the low-salinity range, such as at 10 ppt, on phycoerythrin synthesis has not yet been investigated. Currently, artificial seawater (ASW) is the most common culture medium for *P. purpureum* with a salinity of around 34 ppt maintained by NaCl. The cost of the large-scale cultivation of *P. purpureum* can be reduced if it grows well and accumulates a high content of phycoerythrin under the conditions of low salinity culture medium.

The semi-continuous culture of microalgae is a common cultivation method that replaces part of the culture with an equal volume of fresh medium at specific intervals. This method offers several advantages: (1) it can extend the logarithmic growth phase, maintaining a high growth rate for microalgae; (2) it reduces the negative impact of accumulated metabolites on microalgae growth; (3) it provides the cultivation conditions with adequate nutrients for microalgae [20]. Compared to the continuous culture, the semi-continuous culture is easier to operate, as it does not require complicated equipment for the input and output of the culture medium. In contrast to the batch culture, the semi-continuous culture is a more effective large-scale cultivation strategy, preventing nutrient depletion and harmful metabolite accumulation. A 79 ± 3.4% increase in cell biomass was observed in the semi-continuous cultivation of *Porphyridium cruentum* immobilized in Ca-alginate beads compared with the control [21]. Additionally, recycling the supernatant back into the photobioreactor after harvesting microalgae could further reduce wastewater and the cultivation costs [12,22]. This approach has been successfully applied to the cultivation of *Microchloropsis salina* and *Chlorella sorokiniana* [23,24]. However, since *P. purpureum* secretes large amounts of exopolysaccharides into the culture medium [25], it remains unclear whether reusing such medium would inhibit the growth of *P. purpureum*.

In this study, the effect of salinity on the growth and phycoerythrin accumulation of *P. purpureum* was investigated. Subsequently, two modes of semi-continuous culture were compared by determining the growth and the yield of phycoerythrin, including the recycled culture medium mode and the fresh culture medium mode. This study will provide guidance for the large-scale production of *P. purpureum* to produce phycoerythrin.

## 2. Results

### 2.1. Effect of Salinity on the Growth and Phycoerythrin Content in P. purpureum SCS-02

**Figure 1:**
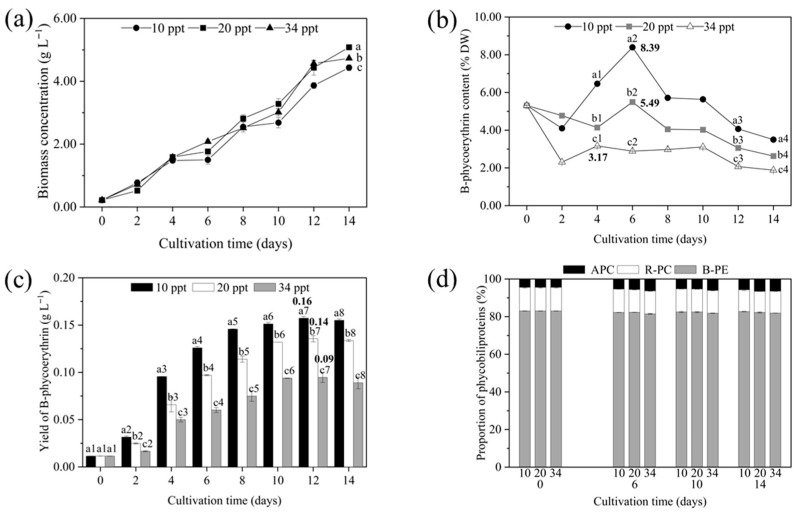
The content of biomass and phycobiliproteins in *Porphyridium purpureum* SCS-02 as a function of time in different salinities: (**a**) biomass concentration; (**b**) phycoerythrin content; (**c**) phycoerythrin yield; (**d**) phycobiliproteins composition. ppt: part per thousand. B-PE: B-phycoerythrin; APC: allophycocyanin; R-PC: R-phycocyanin. The values shown are the averages of two biological replicates and three technical replicates ± standard deviation. Different letters in (**b**) denote significant differences among the phycoerythrin content under different salinities at the same cultivation time (a1–c1: day 4; a2–c2: day 6; a3–c3: day 12; a4–c4: day 14). Different letters in (**c**) denote significant differences among the yields of phycoerythrin under different salinities at the same cultivation time (a1–c1: day 0; a2–c2: day 2; a3–c3: day 4; a4–c4: day 6; a5–c5: day 8; a6–c6: day 10; a7–c7: day 12; a8–c8: day 14) (*p* < 0.05).

The biomass concentrations under different salinity conditions are shown in Figure 1a. Compared to the 20 and 34 ppt salinity groups, the 10 ppt salinity group had lower biomass concentration during the entire cultivation period (*p* < 0.05). The maximum biomass concentration at 10, 20, and 34 ppt salinity conditions reached 4.43, 5.08, and 4.73 g L^−1^, respectively. This reduction at low salinity may be related to osmotic stress, which limits cell growth.

The different salinities had a significant impact on the phycoerythrin content of *P. purpureum* SCS-02. The maximum phycoerythrin content of 10, 20, and 34 ppt salinity groups during the whole cultivation period was 8.39% DW (day 6), 5.49% DW (day 6), and 3.17% DW (day 4), respectively (Figure 1b) (*p* < 0.05). The higher phycoerythrin accumulation at 10 ppt may represent a physiological response to low-salinity stress.

As shown in Figure 1c, the yield of phycoerythrin at 10 ppt salinity was significantly higher than that at 20 and 34 ppt salinity (*p* < 0.05). The maximum phycoerythrin yield for all salinity treatments was observed on day 12, 0.16 g L^−1^ for 10 ppt groups, 0.14 g L^−1^ for 20 ppt groups, and 0.09 g L^−1^ for 34 ppt groups, respectively (*p* < 0.05).

The phycobiliproteins of *P. purpureum* SCS-02 mainly included phycoerythrin, phycocyanin, and allophycocyanin. The proportion of phycobiliproteins of SCS-02 was stable at different salinity groups (Figure 1d). The proportion of phycoerythrin, phycocyanin, and allophycocyanin accounted for approximately 82%, 12%, and 5% of the total phycobiliproteins, respectively. This suggests that salinity in the range of 10–34 ppt did not markedly alter the relative composition of phycobiliproteins.

### 2.2. Semi-Continuous Culture Mode of Recycled Culture Medium

**Figure 2:**
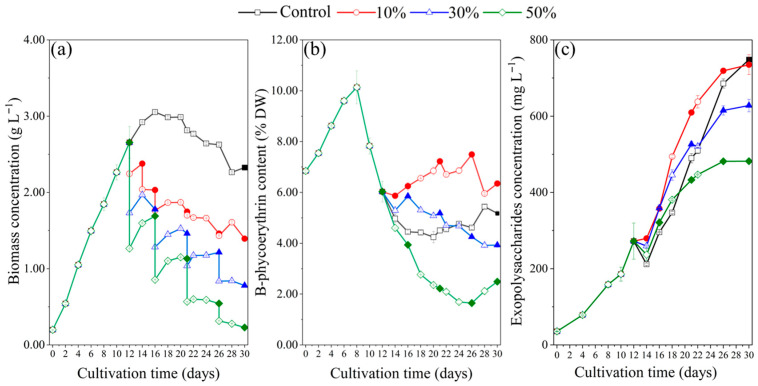
Growth characteristics of *Porphyridium purpureum* SCS-02 under different renewal rates in the recycled culture media: (**a**) biomass concentration; (**b**) phycoerythrin content; (**c**) exopolysaccharides concentration. The values shown are the averages of two biological replicates and three technical replicates ± standard deviation. Control: the batch culture; 10%: renewal rate of 10%; 30%: renewal rate of 30%; 50%: renewal rate of 50%. The filled symbols in the figure represent that the media were renewed on that day. The unfilled symbols represent that no treatment was done on that day.

Starting from day 12, 10%, 30%, and 50% of the culture was harvested, respectively, according to the experimental design described in Section 4.2. Table 2. As shown in Figure 2a, the growth rate of batch culture (the control) slowed down after day 12, and a maximum biomass concentration of 3.05 g L^−1^ was observed on day 16. Harvesting in each RM treatment was performed at the time point when biomass concentration reached its maximum. For the 10% RM, 30% RM, and 50% RM treatments, biomass concentrations increased after the first (day 12), second (day 14), and third (day 16) harvests but stopped rising after the fourth (day 21) and fifth (day 26) harvests. On day 30, the biomass concentrations were 1.39, 0.78, and 0.23 g L^−1^ for the 10% RM, 30% RM, and 50% RM treatments, respectively.

The trend of phycoerythrin content is shown in Figure 2b. The maximum phycoerythrin content of 10.14% DW was observed on day 8 across all groups. In the 30% RM and 50% RM treatments, the phycoerythrin content consistently decreased after each harvest, while the 10% RM treatment showed a slightly increasing trend. Notably, since day 14, the microalgal cells in the 50% RM treatment contained significantly lower phycoerythrin content compared to the control, 10% RM, and 30% RM groups. It suggested that a higher renewal rate negatively impacted phycoerythrin accumulation. However, the phycoerythrin contents in the 10% RM treatment were higher than those in the control, indicating that a 10% renewal rate contributed to phycoerythrin accumulation.

Exopolysaccharides are one of the most important bioactive products in *P. purpureum*. As shown in Figure 2c, the exopolysaccharide concentrations in every group showed an increasing trend from day 14. The maximum exopolysaccharide concentrations for the control, 10% RM, 30% RM, and 50% RM were obtained at day 30, which were 748 mg L^−1^, 735 mg L^−1^, 628 mg L^−1^, and 482 mg L^−1^, respectively. The concentration of exopolysaccharides in the 50% RM treatment was significantly lower than that in the control and other treatments, indicating that microalgal cells cannot recover in time to secrete exopolysaccharides after harvesting at high proportions.

Overall, the results from Figure 2a–c indicate that higher renewal rates of 30% and 50% in the FM mode negatively affected both biomass recovery and the accumulation of phycoerythrin and exopolysaccharides, suggesting that the use of recycled culture medium may impose metabolic stress on the cells.

### 2.3. Semi-Continuous Culture Mode of Fresh Culture Medium

**Figure 3:**
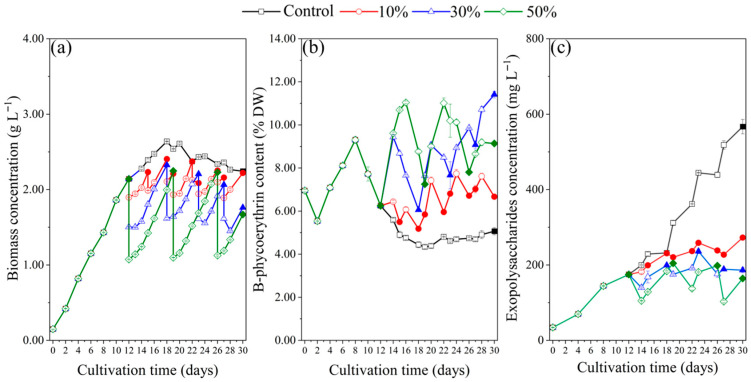
Growth characteristics of *Porphyridium purpureum* SCS-02 under different renewal rates in the fresh culture media: (**a**) biomass concentration; (**b**) phycoerythrin content; (**c**) exopolysaccharide concentration. The values shown are the averages of two biological replicates and three technical replicates ± standard deviation. Control: the batch culture; 10%: renewal rate of 10%; 30%: renewal rate of 30%; 50%: renewal rate of 50%. The filled symbols represent the days with renewal. The unfilled symbols represent the days without renewal.

Beginning on day 12, 10%, 30%, and 50% of the culture was harvested according to the experimental design described in Section 4.2. The changes in biomass concentration are shown in Figure 3a. In the batch culture (the control), the maximum biomass concentration reached 2.64 g L^−1^ on day 18, after which a decreasing trend was observed. For the 10% FM, 30% FM, and 50% FM treatments, harvesting was performed whenever the biomass concentration reached its maximum, whereas the control was harvested only once on day 30. Specifically, harvesting in the 10% FM treatment occurred on day 12, 15, 18, 19, 22, 23, 26, 27, and 30; In the 30% FM treatment on day 12, 18, 23, 27, and 30; and in the 50% FM treatment on day 12, 19, 26, and 30. In all FM treatments, the biomass concentration rapidly recovered to pre-harvest levels after each harvest, indicating that supplementation with fresh medium was beneficial for maintaining a high growth rate of *P. purpureum*.

As shown in Figure 3b, the phycoerythrin content in the batch culture (the control) was significantly lower than that in the semi-continuous culture. The maximum phycoerythrin content in the control was 9.30% DW, observed on day 8. In the 30% FM and 50% FM treatments, their phycoerythrin contents increased quickly after harvesting and the addition of fresh culture medium. For example, the phycoerythrin content increased by 51% in two days in the 30% FM treatment and by 77% in four days in the 50% FM treatment. These results showed that adding fresh culture medium has a positive effect on maintaining high content of phycoerythrin.

In the control, the exopolysaccharides concentration increased continuously from days 12 to 30 at a rate of 22 mg L^−1^ day^−1^, reaching a maximum concentration of 567 mg L^−1^ (Figure 3c). However, there was no significant increase in exopolysaccharides concentration in the 10% FM, 30% FM, and 50% FM treatments, and the mean concentration was 224 mg L^−1^, 184 mg L^−1^ and 158 mg L^−1^, respectively. The high renewal rate generated a low exopolysaccharide concentration.

### 2.4. Comparison of Two Semi-Continuous Culture Modes

**Figure 4:**
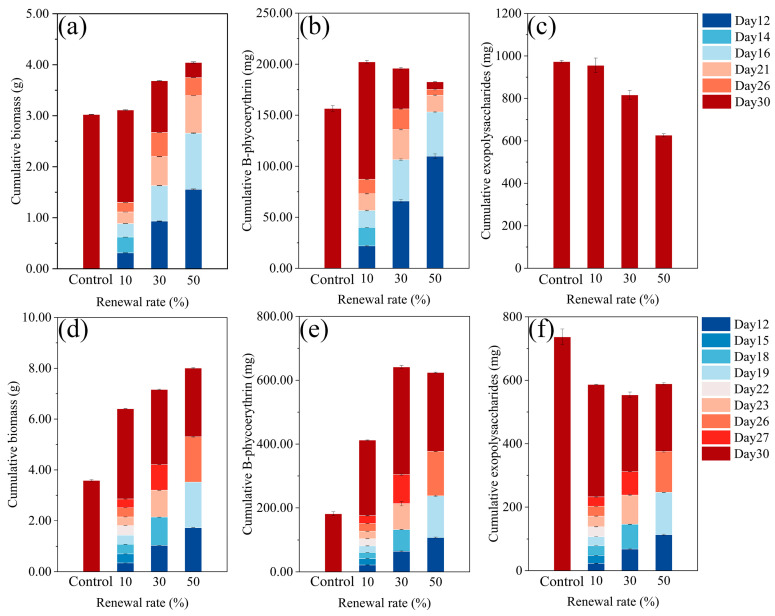
The harvest mass of biomass, phycoerythrin, and exopolysaccharide in *Porphyridium purpureum* SCS-02 under the recycle culture mode (RM) and the fresh culture mode (FM). (**a**–**c**) are the total biomass, phycoerythrin, and exopolysaccharides of the RM mode. (**d**–**f**) are the total biomass, phycoerythrin, and exopolysaccharides of FM mode. The values shown are the averages of two biological replicates and three technical replicates ± standard deviation.

As shown in Figure 4a–c, in the RM mode, the biomass yield in the 10% RM, 30% RM, and 50% RM treatments was 3%, 22% and 34% higher compared to the batch culture (the control). The exopolysaccharide yield in the 10% RM, 30% RM, and 50% RM treatments was reduced by 2%, 16% and 36% compared to the control. The phycoerythrin yield was 29%, 25%, and 17% higher compared to the control.

As shown in Figure 4d, in the FM mode, the biomass yield in the 10% FM, 30% FM, and 50% FM treatments was 79%, 100% and 123% higher compared to the control. The phycoerythrin yield was 127%, 253%, and 244% higher compared to that in the control (Figure 4e). The exopolysaccharide yield in the 10% FM, 30% FM, and 50% FM treatments was reduced by 20%, 25%, and 20%, compared to the control, as shown in Figure 4f.

As shown in Table 1, the total yield of biomass and phycoerythrin was higher in the FM mode than in the RM mode. The maximum yield of biomass and phycoerythrin in the FM mode was 8.01 g (50% FM) and 641.47 mg (30% FM), respectively. The maximum exopolysaccharide yield was 973 mg obtained in the control.

In summary, FM mode markedly enhanced biomass and phycoerythrin yields compared to RM mode, likely due to the continuous supply of fresh nutrients. In both modes, however, exopolysaccharide yields decreased, suggesting that semi-continuous cultivation favors protein accumulation over polysaccharide secretion.

## 3. Discussion

In this study, the growth curves of 10, 20, and 34 ppt salinity treatments indicated that *P. purpureum* SCS-02 could adapt well in the salinity from 10 to 34 ppt (Figure 1a). *Porphyridium* has been widely reported to adapt to a broad range of salinity levels. Among the various strains of *Porphyridium*, *Porphyridium sordidum* can even survive in fresh water. *P. purpureum* SCS-02, which was isolated from the South China Sea with a salinity of 35 ppt, can survive under the salinity of 10 ppt and shows a strong tolerance of low salinity. Culturing *P. purpureum* at low salinity can reduce the cost of medium as well as the desalination of biomass. In addition to affecting the growth of *P. purpureum*, the salinity has a strong influence on phycoerythrin production [26]. In the present study, the highest phycoerythrin content was obtained under low salinity (10 ppt) conditions, suggesting that low salinity might enhance the accumulation of phycoerythrin. This result was similar to the study reported by Lu et al. (2020), which showed that the phycoerythrin content in *P. purpureum* FACHB-806 at 17 ppt salinity was 57% higher than that at 34 ppt salinity [19]. Other microalgae containing phycobiliproteins, such as *Phormidium* sp. and *Cyanobium* sp., also exhibited higher phycobiliprotein content under low salinity conditions compared to high salinity conditions [27,28]. The mechanism by which microalgae respond to changes in salinity stress is encoded in their genes [29]. Low salinity might facilitate the accumulation of phycoerythrin in *P. purpureum* by affecting genes upstream in the phycoerythrin synthesis pathway, such as those involved in regulating nitrogen assimilation. Given the promising results under low salinity of 10 ppt, we further investigated whether cultivation mode, particularly semi-continuous strategies, could improve productivity while reducing cost and environmental impact.

The growth and phycoerythrin synthesis of *P. purpureum* SCS-02 were inhibited in the RM culture mode (Figure 2a). This result was consistent with those observed in other microalgae. For *Arthrospira platensis*, the biomass concentration and phycocyanin content were significantly lower in the 75% RM treatment than in the 25% RM and 50% RM treatments [30]. The biomass concentration and pigment content of *Scenedesmus almeriensis* were greatly reduced in the RM culture mode [31]. *Chlorella vulgaris* showed a faster decrease in growth rate for 40% RM treatment, compared with that for 20% RM treatment [32]. This research demonstrated that the culture medium recycled from *P. purpureum* cannot be reused to culture *P. purpureum*, speculating that the accumulating secondary metabolites in the medium inhibit the growth of *P. purpureum*. The result of this study is consistent with previous studies. Exopolysaccharides are the main organic metabolites present in the aging culture medium, which can cause oxidative damage, inhibiting cell division and pigment accumulation in *Arthrospira platensis* [33,34].

In the FM culture mode, *P. purpureum* SCS-02 maintained a high growth rate and elevated phycoerythrin content under different renewal rates. Over the entire treatment period (days 12−30), the maximum phycoerythrin content reached 7.74% DW on day 24 in the 10% FM treatment, 11.41% DW on day 30 in the 30% FM treatment, and 11.04% DW on day 16 in the 50% FM treatment, all of which were much higher than that of the control (6.25% DW on day 12). During the semi-continuous culture with the fresh culture medium supplementation, *P. purpureum* CCAP 1380/3 had a fast growth rate, as well as a high content of phycoerythrin [25]. Furthermore, the content of phycoerythrin showed a strong negative correlation with the biomass concentration in the FM culture mode. Two possible reasons were speculated: (1) Surface irradiance of microalgal cells might influence the accumulation of phycoerythrin. It has been reported that phycoerythrin content increased when specific irradiance increased from 0.2 to 1.5 W g^−1^ [35], suggesting that high biomass concentrations, which resulted in lower specific irradiance, were unfavorable for phycoerythrin accumulation. (2) Limitation of nutrients inhibited phycoerythrin synthesis. High biomass concentration typically leads to increased consumption of nutrients such as nitrogen. As a nitrogen storage form in red algae, phycoerythrin is more likely to be consumed than synthesized. In this study, there was no significant difference in the yield of exopolysaccharide for 10% FM, 30% FM, and 50% FM, which was consistent with the previous result. In the semi-continuous culture of *Porphyridium* PC-03 with the renewal rates ranging from 10% to 50%, there was no significant difference in the total yield of exopolysaccharides [36]. In addition, the yield of exopolysaccharide was lower in the FM culture mode than that in the batch culture mode, implying FM culture mode was not conducive to the accumulation of exopolysaccharide. A similar result was reported in the previous studies on culturing *Porphyridium* sp. UTEX 637. The higher total polysaccharide content was found in the batch culture rather than in semi-continuous culture [37]. Notably, this contrasts with the enhanced phycoerythrin accumulation observed under the same FM conditions. This difference suggests that the biosynthesis of phycoerythrin and exopolysaccharide may be regulated by distinct physiological signals or metabolic pathways. In the FM culture mode, the replenishment of fresh culture medium reduced the toxic metabolites and provided sufficient nutrition, ensuring active metabolism in the algal cells. However, the mechanism inhibiting exopolysaccharide synthesis under these seemingly favorable conditions remains unclear and requires further research.

From an economic and environmentally friendly perspective, the production of phycoerythrin by the FM culture mode was feasible, with the yield of phycoerythrin increased by 253% compared to the batch culture mode. For example, with a 30-day cultivation cycle in a 1.2 L cultivation system, the yield of phycoerythrin in one time of 30% FM culture mode was 21% higher than that of three times of batch culture mode, while the medium consumption was reduced by 27%. Another important consideration is that, in semi-continuous culture, the presence of exopolysaccharides can reduce the efficiency of cell harvesting due to their relatively high viscosity [38,39]. These polymers make solid–liquid separation difficult when using membrane filtration or centrifugation, as also reported in previous studies [40] and confirmed by our observations. In the FM mode, however, the mean concentration of exopolysaccharides (Figure 3c) was much lower than that in batch culture, which may facilitate cell harvesting and lower the energy demand of the process. However, in the RM mode, the yield of phycoerythrin is slightly higher than that of the batch mode. Unless an economical and efficient method for removing exopolysaccharides from the recycled medium can be developed, the RM mode is not feasible. For the production of exopolysaccharides, since there was no significant difference in the yield between the 10% RM mode and the batch mode, the batch culture appears to be the better approach based on the results of this study.

Taken together, these findings highlighted the potential of low salinity and semi-continuous cultivation strategy to enhance phycoerythrin production in *P. purpureum*. A limitation of this study was that only two biological replicates were used in each cultivation condition. Although three or more biological replicates are desirable for greater statistical power, the large scale (1.2 L working volume) and long duration (30-day semi-continuous operation) of the experiments prohibited the inclusion of additional replicates in this study. Nevertheless, the two biological replicates showed highly consistent trends, and the results still offer informative and valuable reference points for understanding the cultivation performance of *P. purpureum*.

## 4. Materials and Methods

### 4.1. Strain and Culture Conditions

*Porphyridium purpureum* SCS-02, isolated from the South China Sea, was used as an experimental microalga strain [4]. *P. purpureum* SCS-02 was cultured in standard glass column photobioreactors (Φ6 cm × 60 cm) containing 1.2 L of ASW culture medium, as described by Li et al. [41]. The ASW medium consisted of 17.6 mM NaNO_3_, 2.07 mM K_2_HPO_4_, 0.48 mM NaHCO_3_, 11.7 µM EDTANa_2_·2H_2_O, 11.7 µM FeCl_3_·6H_2_O, 0.91 µM MnCl_2_*·*4H_2_O, 0.08 µM ZnSO_4_·7H_2_O, 0.02 µM Na_2_MoO_4_·2H_2_O, 0.04 µM CoCl_2_·6H_2_O and 0.04 µM CuSO_4_·5H_2_O. The compressed air with 1% CO_2_ (Air: CO_2_, 99:1) was continuously bubbled in the photobioreactor. The culture temperature was maintained at 25 ± 1 °C. A bank of single-sided T8 fluorescent lamps (Philips, Amsterdam, The Netherlands) was used for irradiation, with the light intensity at 130 μmol photons m^−2^ s^−1^. The photoperiod was 24 h: 0 h (light: dark). The initial OD_750_ (the optical density of 750 nm) was 0.30 ± 0.02, measured using the TU-1810 UV spectrophotometer (Persee Instrument Co., Ltd., Beijing, China).

### 4.2. Experimental Design

Salinity: Three treatments with different salinities were set up, including 10 parts per thousand (ppt), 20 ppt, and 34 ppt, respectively. The concentration of biomass and phycoerythrin content at different salinities was measured during the cultivation period.

Culture modes: The salinity of subsequent experiments was determined by the result of initial salinity experiments.

(1) The control: The batch culture was conducted for 30 days. Every two days, samples were harvested for the measurement of biomass, phycoerythrin, and exopolysaccharide content.

(2) Recycled culture medium (RM) mode: Three treatment groups with recycled culture medium were set up, including 10% RM, 30% RM, and 50% RM. The biomass concentration of each treatment was measured daily. When the biomass concentration reached its maximum, 10%, 30% and 50% of the culture were harvested by centrifugation (8000 rpm for 10 min), and the supernatant was returned to the photobioreactor. The contents of biomass, phycoerythrin, and exopolysaccharides were measured, and the yields were calculated. NaNO_3_ and K_2_HPO_4_ were supplemented to the culture medium on days 6, 12, 18, and 24 to ensure adequate nutrition of nitrogen and phosphorus.

(3) Fresh culture medium (FM) mode: Three treatment groups using fresh culture medium were established, including 10% FM, 30% FM, and 50% FM. The biomass concentration of each group was measured daily. When the biomass concentration reached its maximum, 10%, 30% and 50% of the culture, respectively, was harvested by centrifugation (8000 rpm for 10 min), and an equal volume of fresh culture medium was added to the photobioreactor. The contents of biomass, phycoerythrin, and exopolysaccharide were measured, and the yields were calculated.

The specific harvesting schedules for each treatment group are summarized in Table 2.

### 4.3. Growth Measurement

The 10 mL culture was filtered through a 0.45 μm pre-weighed filter membrane (Tianjin Jinteng Experimental Equipment Co., Ltd., Tianjin, China). The filters were dried at 80 °C for 5 h and reweighted. The biomass concentration was measured before harvesting and remeasured after adding either recycled or fresh culture medium.

### 4.4. Determination of Phycobiliproteins Content

The 5 mL of Tris-HCl buffer (20 mM, pH 8.0) was added to the wet biomass for repeated freeze-thawing at −20 °C and 4 °C for 24 h. The supernatant was collected by centrifugation at 8000 rpm for 10 min. The absorbance at the wavelengths of 565, 620, and 650 nm was measured by a TU-1810 UV spectrophotometer. The phycobiliproteins content was calculated by the following Equations (1)−(7) [42]:

C_R-PC_ = (OD_620_ − 0.7 × OD_650_)/7.38, (1)

C_APC_ = (OD_650_ − 0.19 × OD_620_)/5.65, (2)

C_B-PE_ = (OD_565_ − 2.8 × C_R-PC_ − 1.34 × C_APC_)/12.7 (3)

R-PC (%DW) = (C_R-PC_ × 5)/(M × V) × 100% (4)

APC (%DW) = (C_APC_ × 5)/(M × V) × 100% (5)

B-PE (%DW) = (C_B-PE_ × 5)/(M × V) × 100% (6)

The yield of B-PE (g L^−1^) = B-PE (%DW) × M (7)

where C_R-PC_, C_APC_, and C_B-PE_ are the contents of R-phycocyanin, allophycocyanin, and B-phycoerythrin, respectively (mg mL^−1^). OD_565_, OD_620_, and OD_650_ are the absorbance at 565 nm, 620 nm, and 650 nm, respectively. M is the biomass concentration (g L^−1^). V is the volume of the extract solution (L).

### 4.5. Determination of Exopolysaccharide Content

The aliquot of the culture was centrifuged at 8000 rpm for 10 min. The supernatant was collected and stored at −20 °C for the determination of exopolysaccharides concentration. The content of exopolysaccharide was measured using the phenol-sulfuric acid method [43]. If the exopolysaccharide concentration was too high, the supernatant was diluted to the proper concentration.

### 4.6. Statistical Analysis

The means and standard deviations presented in all figures and tables were calculated from two biological replicates with three technical replicates each, conducted under strictly controlled conditions. Owing to the large scale of the cultivation system and the associated resource constraints, all experiments were limited to two biological replicates and three technical replicates. Statistical analyses were performed using SPSS 18.0 software (SPSS Inc., Chicago, IL, USA). One-way analysis of variance (ANOVA) was used to assess the significance of differences among multiple treatment groups. The least significant difference (LSD) at α = 0.05 was used to determine statistically significant differences between treatments.

## 5. Conclusions

*P. purpureum* SCS-02 was able to grow under salinities of 10, 20, and 34 ppt, with the highest content and yield of phycoerythrin observed at 10 ppt. Reusing the culture medium recycled from *P. purpureum* cultivation inhibited the growth and the phycoerythrin accumulation. The yield of phycoerythrin in the semi-continuous culture mode with the 30% renewal rate of fresh culture medium was increased by 253% compared with the batch culture. However, exopolysaccharide yields were significantly reduced in the semi-continuous culture with fresh medium supplementation relative to batch culture.

## Figures and Tables

**Table 1:** The yield of biomass, phycoerythrin, and exopolysaccharides in *Porphyridium purpureum* SCS-02 under different renewal rates after 30 days of cultivation.

	Biomass Yield (g)		Phycoerythrin Yield (mg)		Exopolysaccharides Yield (mg)	
Renewal Rate	RM	FM	RM	FM	RM	FM
Control	3.30 ± 0.31 ^b1,d2^		169.08 ± 14.92 ^b3,c4^		854.9 ± 137.09 ^a5,a6^	
10%	3.11 ± 0.00 ^b1^	6.41 ± 0.00 ^c2^	202.22 ± 1.49 ^a3^	412.23 ± 1.67 ^b4^	955.62 ± 34.27 ^a5^	586.52 ± 2.39 ^b6^
30%	3.69 ± 0.01 ^a1^	7.16 ± 0.02 ^b2^	195.94 ± 3.52 ^a3^	641.47 ± 12.73 ^a4^	816.11 ± 21.22 ^a5,b5^	553.81 ± 10.05 ^b6^
50%	4.04 ± 0.02 ^a1^	8.01 ± 0.01 ^a2^	182.71 ± 2.34 ^a3,b3^	624.05 ± 1.08 ^a4^	626.73 ± 6.53 ^b5^	588.95 ± 7.30 ^b6^

The values shown are the averages of two biological replicates and three technical replicates ± standard deviation. Different letters denote significant differences among the biomass yield, phycoerythrin yield and exopolysaccharide yield under different renewal rates (level of significance, *p* < 0.05) (^a1–b1^ and ^a2–d2^: the biomass yield of RM and FM; ^a3–b3^ and ^a4–c4^: the phycoerythrin yield of RM and FM; ^a5–b5^ and ^a6–b6^: the exopolysaccharides yield of RM and FM). RM: recycle culture medium mode; FM: fresh culture medium mode.

**Table 2:** Summary of the harvesting schedule for each treatment.

	Renewal Rate				Renewal Rate		
Day	RM	FM	Control	Day	RM	FM	Control
0	NT	NT	NT	16	10%, 30%, 50%	NT	NT
1	NT	NT	NT	17	NT	NT	NT
2	NT	NT	NT	18	NT	10%, 30%	NT
3	NT	NT	NT	19	NT	10%, 50%	NT
4	NT	NT	NT	20	NT	NT	NT
5	NT	NT	NT	21	10%, 30%, 50%	NT	NT
6	NT	NT	NT	22	NT	10%	NT
7	NT	NT	NT	23	NT	10%, 30%	NT
8	NT	NT	NT	24	NT	NT	NT
9	NT	NT	NT	25	NT	NT	NT
10	NT	NT	NT	26	10%, 30%, 50%	10%, 50%	NT
11	NT	NT	NT	27	NT	10%, 30%	NT
12	10%, 30%, 50%	10%, 30%, 50%	NT	28	NT	NT	NT
13	NT	NT	NT	29	NT	NT	NT
14	10%	NT	NT	30	100%	100%	100%
15	NT	10%	NT				

NT: No treatment; RM: recycled culture medium mode; FM: fresh culture medium mode; 10%: renewal rate of 10%; 30%: renewal rate of 30%; 50%: renewal rate of 50%.

## Data Availability

Data is contained within the article: The original contributions presented in this study are included in the article. Further inquiries can be directed to the corresponding author.
